# A case report of pelviscopic resection of invasive hydatidiform mole

**DOI:** 10.1097/MD.0000000000017565

**Published:** 2019-10-11

**Authors:** Hyun Joo Lee, Yun Sook Kim

**Affiliations:** a Department of Pathology; b Obstetrics and Gynecology, Soonchunhyang University College of Medicine, Soonchunhyang University Cheonan Hospital, Cheonan, Republic of Korea.

**Keywords:** beta-human chorionic gonadotropin, invasive mole, pelviscopy, transvaginal ultrasound

## Abstract

**Rationale:**

Invasive moles occur in the fertile period, with about 95% occurring after previous mole removal and the remaining 5% occurring after several other pregnancies.

**Patient concerns:**

A 27-year-old patient developed a rare invasive mole two months after a missed abortion.

**Diagnoses:**

A transvaginal ultrasound scan revealed a 3.6 × 2.9 × 2.4 cm sized lesion with cystic vascular areas within it, within the myometrium of the right fundal posterior region of the uterus. There was no metastasis to other organs.

**Interventions:**

After administration of methotrexate, the level of beta-human chorionic gonadotropin (ß-hCG) was elevated and liver enzymes were also markedly elevated. She wanted to retain fertility for future pregnancies. After laparoscopic removal of the myometrial invasive mole, the incision site was sutured with a 3-0 V-Loc^TM^.

**Outcomes:**

One year later, a natural pregnancy occurred and a cesarean section was performed at 36 weeks.

**Lessons:**

This is the first reported case of its type. Our case demonstrated that pelviscopic removal of an invasive mole is possible if there are no other metastases, and that future pregnancy and childbirth are still feasible in women of reproductive age.

## 1 Introduction

Invasive H-moles or invasive moles are a type of abnormal pregnancy that grows into the muscular wall of the uterus. It may aggressively invade the myometrium and may even perforate the uterine serosa. The most common life-threatening complication of invasive moles is uterine perforation. After rupture of the uterus, massive bleeding, hemoperitoneum, and acute abdominal pain occurs. ^[1]^ Invasive moles occur mainly in women of child-bearing age, and rarely occurs in perimenopausal women.^[2]^ Ultrasonography corresponds with the classical clinical presentation and an accurate diagnosis is possible in a majority of cases. With early and accurate diagnosis, the cure rate is high.^[3]^

## 2 Case report

**Figure 1:**
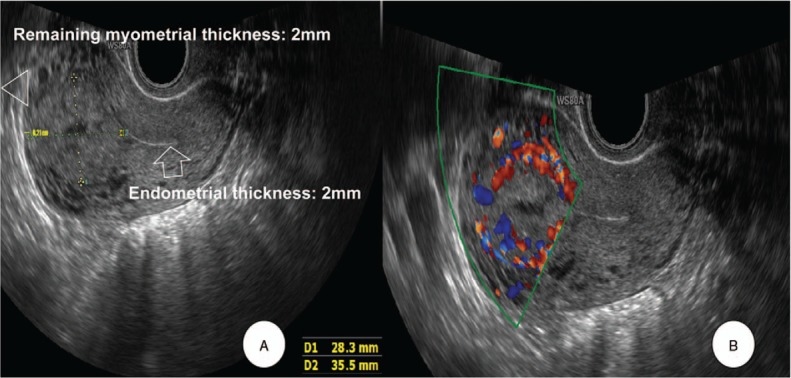
Transvaginal ultrasound. (A) Brightness-mode showed 2 mm endometrial thickness (arrow). A 3.6 × 2.9 × 2.4 cm sized heterogenous hyperechoic solid lesion with cystic vascular areas within it, was noted within the myometrium of the right fundal posterior region of the uterus, away from the endometrium. The thickness of the remaining muscle layer with invasion of the invasive mole was very thin, 2 mm (arrowhead). (B) Color Doppler showed prominent blood flow signals of various directions of arterial and venous flows within the lesion, which were also seen within the cystic areas of the lesion.

**Figure 2:**
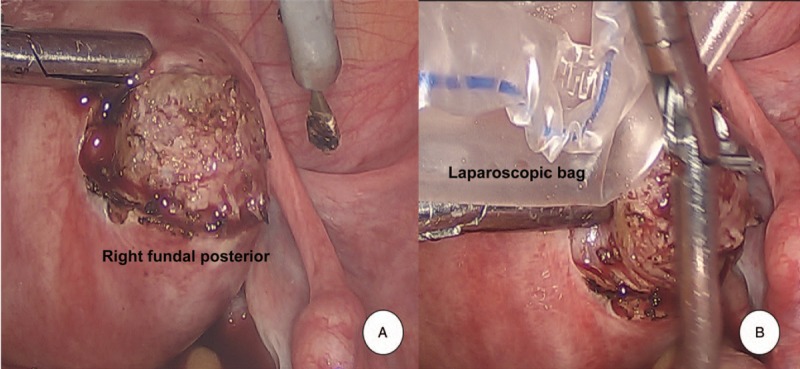
Pelviscopic findings. (A) In the abdominal cavity, the right fundal posterior wall was protruded. After incision of the protruding site, invasive mole was observed. (B) Tissues of the invasive mole were removed with laparoscopic bag to prevent seeding. The incision site was sutured with a 3-0 V-Loc^TM^ device.

**Figure 3:**
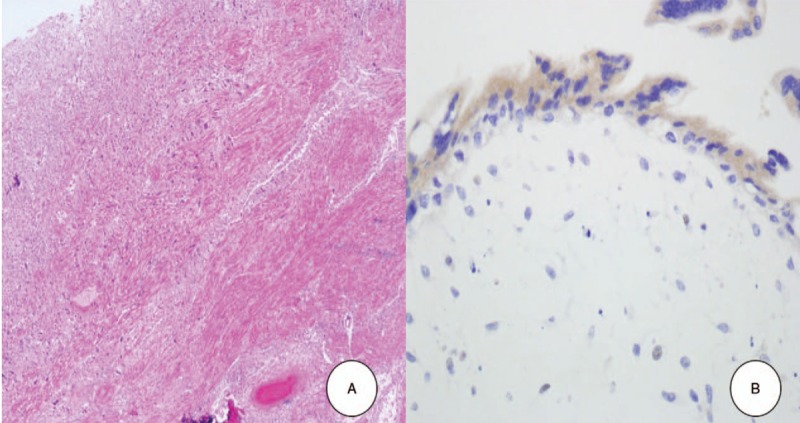
Microscopic examinations. (A) Distended hydropic villi with circumferential trophoblastic proliferation was noted (Hematoxylin-eosin, ×40). (B) Trophoblastic cells exhibited moderate to severe cellular pleomorphism and immunohistochemical staining for p57 was negative in cytotrophoblasts (×400).

A 27-year-old Asian female, gravida 1, para 0, presented to our department with amenorrhea of six months and elevated beta-human chorionic gonadotropin (ß-hCG) levels. Her physical examination was unremarkable. Her gynecological history was notable for the occurrence of an abortion at 12 weeks gestation six months previously, which turned out to be a missed abortion. She subsequently underwent two suction and curettage procedures, as the ß-hCG was persistently elevated to 50,063 mIU/ml. Following this, however, the ß-hCG still continued to rise, reaching 17,008 mIU/ml. The patient underwent two more dilatation and curettage procedures and was given an injection of 50 mg methotrexate around one month prior to presentation at local clinic. Menstruation had still not returned. Serial ß-hCG tests were performed, and levels initially fell to 68 mIU/ml, then remained constant around 60 mIU/ ml for eight weeks, at which time she was referred to our hospital. A transvaginal ultrasound scan revealed that both ovaries were unremarkable. Endometrial thickness was 2 mm. A 3.6 × 2.9 × 2.4 cm sized heterogenous hyperechoic solid lesion with cystic vascular areas within it, was noted within the myometrium of the right fundal posterior region of the uterus, away from the endometrium. The thickness of the remaining muscle layer with invading mole was very thin, 2 mm (Fig. 1A). The color Doppler showed prominent blood flow signals of various arterial and venous flow directions within the lesion, which were also seen within the cystic areas of the lesion (Fig. 1B). No evidence of an intrauterine gestational sac or ectopic pregnancy was noted. Based on the clinical history of missed abortion in the past, with persistently elevated ß-hCG levels, a sonographic diagnosis of an invasive mole of the uterus was established. A complete metastatic work-up was done which did not reveal any metastasis. The patient was started on 50 mg methotrexate immediately. The next day, however, her ß-hCG level rose, liver enzyme levels rose dramatically, and she complained of nausea. The patient had no children, but wanted to be able to sustain a pregnancy and bear a child. After consultation with the patient, we decided to do a pelviscopic resection of the invasive mole. When we entered the abdominal cavity, the right fundal posterior wall protruded. That site was incised, and invasive mole was observed (Fig. 2A). After removing the mole from the abdominal cavity by bagging, the suture was sealed with a 3-0 V-Loc^TM^ absorbable wound closure device (Medtronic Co. Minneapolis, MN, USA) (Fig. 2B). Microscopic examination revealed the placental tissue had avascular hydropic villi with a central cistern and circumferential proliferation of trophoblastic cells, which invaded the myometrial wall and vessels (Fig. 3A). The trophoblastic cells showed cytologic atypia and immunohistochemical staining for p57 was negative in cytotrophoblastic cells (Fig. 3B), suggesting complete hydatidiform mole. On the first day of surgery, the ß-hCG was reduced to 23 mIU/ml, and on the second day of surgery it had decreased to 8 mIU/ml, and she was discharged. After one week of discharge, the level dropped to 0.7 mIU/ml. A repeat transvaginal ultrasound and color Doppler scan was done a month later, and did not reveal any abnormality, suggesting complete response to therapy. One year later, she underwent a normal natural pregnancy and cesarean delivery at 36 weeks of gestation. Placenta had no abnormalities in pathology. We obtained informed consent to publish from the patient.

## 3 Discussion

Invasive hydatidiform moles develop in approximately 10% to 20% of patients after molar evacuation and infrequently after other gestations. Due to their aggressive growth characteristics, invasive moles are considered locally invasive non-metastatic neoplasms. They are defined as moles that penetrate and may even perforate the uterine wall. The tumor is locally destructive and may invade parametrial tissue and blood vessels, eventually leading to hemoperitoneum. ^[4]^ It is important to distinguish between invasive moles and choriocarcinoma, as the former has a more favorable outcome. The clinical presentation of an invasive mole includes vaginal bleeding, an enlarged uterus, and high urinary or serum ß-hCG levels, typically after the evacuation of a molar pregnancy. The interval from an antecedent molar pregnancy is usually less than six months. Choriocarcinoma can occur after a hydatiform mole or even after a normal pregnancy, with an interval of more than 6 months, sometimes lasting for nearly 10 years. ß-hCG levels are much higher in choriocarcinoma than in invasive moles.^[5]^ Approximately 8% of patients with complete moles will develop a malignant tumor after evacuation.^[6]^ The Cancer Committee of the International Federation of Gynaecologists and Obstreticians (FIGO) has established the following guidelines for the diagnosis of post molar gestational trophoblastic neoplasia (GTN):^[7]^ four values or more of ß-hCG plateaued over at least 3 weeks or an increase in ß-hCG of 10% or greater for 3 or more values over at least 2 weeks. Ultrasound has become the standard protocol employed to assist in diagnosis of suspected GTN.^[8]^ Brightness-mode ultrasound is useful to detect the presence of abnormal uterine masses. Sonographically, an invasive hydatidiform mole typically exhibits a heterogenous, hyperechoic, solid mass with cystic vascular spaces, located within the myometrium.^[9]^ Color Doppler imaging aids in the assessment of angiogenesis and neovascularization characteristic in these tumors, seen as prominent blood flow signals in various directions, suggestive of arterial and venous flow. Demonstration of a vascular mass within the myometrium on ultrasonography, without evidence of fetal material, in the context of an elevated ß-hCG is highly suggestive of GTN. Its pathology is characterized by the presence of edematous chorionic villi with trophoblastic proliferation that invades into the myometrium of the uterus. ^[10]^ Invasive moles are rarely metastatic, but if metastasis does occur, it is usually to the lungs. Management of an invasive mole includes treatment with chemotherapy, as well as continued monitoring of ß-hCG. Patients with GTN should be followed with weekly quantitative ß-hCG levels until normal for three consecutive weeks, then monthly for 12 months.^[11]^ Dilatation and curettage is not recommended due to the risk of uterine perforation. With methotrexate, complete remission is achieved in most non-metastatic and low risk cases.^[12]^ One study reported laparoscopic removal of a corneal invasive mole and the patient was administered methotrexate chemotherapy.^[13]^ After laparoscopic myometrial resection, pregnancy should be avoided for at least 6 months, and a cesarean section is necessary. The value of this case report can be seen in the following three points. First, in general, more than 95% of invasive moles occur after a previous mole removal, and very rarely occur after a missed abortion. Second, in the absence of distant metastasis, treatment with methotrexate is appropriate. However, the patient had severe adverse drug reactions, pelviscopic resection of the invasive mole site for future pregnancy. Third, 1 year later, a natural pregnancy occurred and a cesarean section was performed at 36 weeks without uterine rupture. No such case has ever been reported. It was accurately diagnosed by transvaginal ultrasound and color Doppler, and successfully treated by pelviscopic resection before any major complications could arise. We suggest that this rare valuable case is likely to be of help to many clinicians.

## Author contributions

**Conceptualization:** Yun Sook Kim.

**Data curation:** Yun Sook Kim.

**Investigation:** Yun Sook Kim.

**Writing – original draft:** Yun Sook Kim.

**Writing – review & editing:** Hyun Joo Lee.

Yun Sook Kim orcid: 0000-0001-8427-4006.
